# ﻿Original descriptions of Palaearctic species of the genus *Plateumaris* C. G. Thomson, 1859 (Coleoptera, Chrysomelidae, Donaciinae) and their translations

**DOI:** 10.3897/zookeys.1177.103212

**Published:** 2023-08-30

**Authors:** Elisabeth Geiser, Remigius Geiser

**Affiliations:** 1 Associate Scientist at Natural History Museum, Burgring 7, 1010 Vienna, Austria Associate Scientist at Natural History Museum Vienna Austria; 2 Saint-Julien-Strasse 2/314, 5020 Salzburg, Austria Unaffiliated Salzburg Austria

**Keywords:** French descriptions, German descriptions, Latin descriptions, leaf-beetles, Palaearctic Region, reed-beetles, Russian descriptions, taxonomy

## Abstract

Many original descriptions of beetles were published in Latin with specific idioms and technical terms, which are sometimes difficult to understand. The exact meaning of these descriptions is necessary for taxonomic and systematic research. Of the ten Palaearctic *Plateumaris* species regarded as valid three were described in English, the remaining seven in Latin, French, or German: *P.amurensis* Weise, 1898, *P.bracata* (Scopoli, 1772), *P.consimilis* (Schrank, 1781), *P.roscida* Weise, 1912, *P.rustica* (Kunze, 1818), *P.sericea* (Linnaeus, 1758), and *P.weisei* (Duvivier, 1885). These seven non-English original descriptions and their translations into English are presented here. Additionally, the translations of the first descriptions of the genus *Plateumaris* and of its 19 synonyms (some were described in Russian, also) are given.

## ﻿Introduction

Since Linnaeus (1758) many animal taxa have been described in Latin with very specific phrases used in scientific entomological scripts in the 18^th^ and 19^th^ centuries, which are not easily understandable for classicists who are not also entomologists. Furthermore, knowledge of the Latin language is in rapid decline. It is no longer part of the education curriculum of grammar schools, but was mandatory in Europe for centuries. Nowadays, English is the worldwide communication language used in science. The second author is a classicist and an entomologist with profound knowledge in systematics who translated numerous original Latin descriptions of Donaciinae taxa in the last years for systematic studies by the first author, such as *Donaciaclavareaui* Jacobson, 1906 (Geiser 2019). Many translations are yet unpublished, but were used in Geiser and Jäch (2021).

First descriptions were also given in German, French, or Russian. These languages can be translated by electronic tools into understandable, but not always into directly printable English. However, these means do not offer the possibility to translate Latin into English. The Latin language was used for texts for more than 2000 years, for different purposes: in theology, law, medicine, philosophy, and scientific texts up to the 20^th^ century, and in all these topics with different grammar and vocabulary rules. Even the Latin descriptions of beetles vary between beetle families. Also, they depended upon the specific education and on the preference of the authors. Therefore, “hand-made” or better “brain-made” translations by a knowledgeable specialist will provide the most adequate results.

In the update of the Catalogue of Palaearctic Chrysomelidae – Donaciinae (Geiser and Bezděk in press; Geiser in press) the first author made many changes concerning the taxonomy and systematics of the *Plateumaris* species compared with the first edition of the catalogue (Silfverberg 2010). These changes are explained in Geiser (2023). For such systematic revisions it was essential to study not only many specimens, but also the original descriptions.

In the genus *Plateumaris* 80 taxa were described for the Palaearctic region. Ten species names are regarded as valid, the other 70 are synonyms. We provide here the translations of the original descriptions of seven valid species (the other three were described in English) of the genus *Plateumaris*, and, additionally, of 19 taxa which are regarded as synonyms. These taxa are discussed in detail in Geiser (2023). Some original descriptions in other languages than Latin or English were also translated here into English. All these texts were essential for arguments why some systematic changes, especially synonymisations, were made in Palaearctic *Plateumaris* species.

## ﻿Material and method

All original descriptions published in Latin were translated into German by the second author. Then the first author translated them into English. There also exist some original descriptions in German, which were translated into English by the first author. Some original descriptions were published in French, Russian, and Serbian. They were first translated into English by Google translate. Then the translations of French texts were revised together with Gloria Geiser. Translations of texts in a Slavic language were checked with Irmgard Geiser. Some original descriptions are multilingual. They begin in Latin, but often more details are explained in the author’s native language. The original orthography of the description is printed here, even though it is now outdated; the translation into English is as accurate and faithful as possible to the original text. The names of the *Plateumaris* taxa are arranged alphabetically, regardless of whether they are now synonyms or names of a valid species. The Palaearctic species *P.akiensis* Tominaga & Katsura, 1984, *P.constricticollis* Jacoby, 1885, and *P.shirahatai* Kimoto, 1971 are not included here because they were originally described in English.

Text in square brackets contains additions by the authors and is not part of the original description.

## ﻿Results

### ﻿*Plateumaris* Thomson, 1859

Described on page 154.

**Table:** 

**Latin**	**English**
**Familia Donaciidæ**	**Family Donaciidae**
Antennæ ante oculos insertæ. Caput exsertum, paullo pone oculos constrictum. Elytra striata, striola suturali abbreviata. Coxæ posticæ late distantes. Tibiæ calcari obsoleto. Abdomen segmento 1:o ceteris simul sumtis longitudine æquali.	Antennae inserted in front of the eyes. Head prominent, slightly constricted behind the eyes. Elytra striated, sutural stripe shortened. Posterior coxae far apart. Tibiae with an inconspicuous spur. 1^st^ abdominal segment as long as the others put together.
*Donacia* Fab. Gyll. Typus *D.crassipes* (Fab.):	*Donacia* Fab. Gyll. Typus *D.crassipes* (Fab.): Gyll. III. 646. 1.
Gyll. III. 646. 1.	Hind margin of the tibiae to some extent carinate. Eyes large, slightly protruding. Mandibles short.
Tibiæ margine postico subcarinato. Oculi magni, prominuli. Mandibulæ breves.	
*Plateumaris*. Donacia Gyll. Typus *P.nigra* (Fab.): Gyll. IV. 678. 10–11. Tibiæ multi-angulatæ. Femora breviora, crassa. Mandibulæ validæ, labrum longe superantes. Antennae inter se non magis quam ab oculis parvulis distantes.	*Plateumaris*. Donacia Gyll. Typus *P.nigra* (Fab.): Gyll. IV. 678. 10–11. Tibiae polygonal. Femora shorter, thick. Strong mandibles, well overlapping the labrum. Antennae not further distant from each other than from the small eyes.
*Hæmonia* Latr. Donacia Gyll. Typus H. Zosteræ (Fab.): Gyll. IV. 683. 17.	*Haemonia* Latr. Donacia Gyll. Typus H. Zosterae (Fab.): Gyll. IV. 683. 17.
Tarsi articulo ultimo ceteris simul sumtis longiore. Elytra apice spinoso-producta. Antennæ basi subcontiguæ.	Last [sic!] segment of the tarsi longer than the others put together. Elytra extended thorn-like at the apex. Antennae to some extent close together at base.

### ﻿*Plateumarisaffinis* (Kunze, 1818)

Described as *Donaciaaffinis* on page 37. Synonym of *P.rustica*.

**Table:** 

**Latin**	**English**
***Donaciaaffinis***	***Donaciaaffinis***
*D.affinis*; thorace elongato subquadrato, depresso, subimpresso, punctato, angulis anticis rotundatis, lateribus subsinuatis; elytris convexiusculis, subtilissime punctulato-rugulosis, apice rotundatis, punctis striarum levibus, discretis, striis remotiusculis, pedibus pallescentibus.	*D.affinis*; the elongated pronotum almost square, flat, slightly depressed, punctured, front corners rounded, sides slightly convex; elytra slightly arched, extremely finely punctate and wrinkled, rounded at the end, the points of the stripes are faint, separated, the stripes are a little bit distant, legs slightly paler.
*D.nigra* Payk. Fn. Suec. II. p. 196. 10.	*D.nigra* Payk. Fn. Suec. II. p. 196. 10.
*D.discolor* Gyll. Ins. Suec. III. 660. 10. (teste Zetterstedt.)	*D.discolor* Gyll. Ins. Suec. III. 660. 10. (teste Zetterstedt.)
*D.fusca* Mus. Lesk. nr. 591 ?	*D.fusca* Mus. Lesk. nr. 591 ?
*D.simplex* Thunberg. N. Act. Ups. V. p. 118. 55.	*D.simplex* Thunberg. N. Act. Ups. V. p. 118. 55.
*L.fusca* Gmel. L. Syst. Nat. 18. 67. 86. 2. ?	*L.fusca* Gmel. L. Syst. Nat. 18. 67. 86. 2. ?

### ﻿*Plateumarisamurensis* Weise, 1898

Described on page 179. The German part of the text is shown in the original but the now-outdated orthography.

**Table:** 

**Latin**	**English**
***Plateumarisamurensis***	***Plateumarisamurensis***
Oblonga, convexiuscula, obscure aenea, subtus cinereo-sericea, abdominis segmentis postice, ano, pedibus, ore antennisque dilute ferrugineis, his brevibus, articulo 3: o: 2: o longiore,	Long, slightly convex, dark ore-coloured, underside silky grey, the abdominal segments from behind, anus, legs, mouth, and antennae are pale reddish brown, these [the antennae] are short, the third antennomere is longer than the second one, [3: o: 2: o is surely an error of the typesetter, presumably caused by misunderstanding of the [handwritten?] manuscript; the correct Latin description is: “articulo tertio secundo longiore” or “articulo 3° 2° longiore” or “articulo 3o: 2o longiore”]
prothorace brevi subcordato, disco dense ruguloso-punctato, subopaco, basi profunde arcuatim impresso, canalicula media sat profunda, angulis anticis minimis, acutis, elytris apice rotundato-truncatis, striato-punctatis, interstitiis nitidis, transversim strigosis, femoribus posticis dente valido armatis.	the short prothorax subcordate, the disc is rugose and densely dotted, almost dark, at the basis a deep arcuate impression, the middle groove rather deep, anterior angles very small and acute, elytra at apex rounded truncate, with rows of punctures, intervals shiny, lean in transverse direction, hind femora armed with a stout tooth.
Long 7,5 mm.	Length 7.5 mm
Amur	Amur [location of the type specimen]
**German**	**English**
Mit *Pl.discolor* verwandt, Fühler und Halsschild kürzer, letzteres viel unebener, erstere nebst den Mundtheilen, dem Hinterrande der Bauchringe und dem Aftersegmente hell rostroth.	Related to *Pl.discolor*, antennae and pronotum shorter, the latter much more uneven, the former together with the mouth parts, the posterior margin of the abdominal segments and the anal segment pale rusty red.
Halsschild etwas breiter als lang, die Vorderecken bilden einen kleinen, aber scharfen Borstenkegel, die Seiten sind dahinter gerundet-erweitert, von ¼ der Länge ab allmählich nach hinten verengt, in der Mitte sanft ausgeschweift. Die Oberfläche ist uneben, dicht runzelig punktirt und mehr glänzend; sie fällt nach innen sanft zur scharfen Mittelrinne ab, die vor der Basis in einem tiefen, bogenförmigen Quereindrucke endet. So entstehen auf jeder Seite 2 niedrige Beulen, die vordere derselben ist kleiner als die hinter der Mitte.	Pronotum slightly wider than long, the anterior angles form a small but sharp conical edge with bristles, behind it the sides are rounded-expanded, gradually narrowing backwards from ¼ of length, gently flared in the middle. The surface is uneven, densely rugose dotted and more lustrous; it slopes gently inwards to the sharp median groove, which ends with a deep, arched transverse impression before the basis. This creates 2 low tubercles on each side, of which the anterior one is smaller than the one behind the middle.
Die Flügeldecken sind wie bei den schwach sculptirten Exemplaren von *discolor* punktirt, die Punkte scharf eingestochen, die Zwischenstreifen glänzend, fein quer gerunzelt. Halsschild und Flügeldecken sind schwarzbraun, mit einem starken Messingschimmer.	The elytra are punctured like the weakly sculptured specimens of *discolor*, the points are sharply engraved, the intervals are shiny and finely wrinkled transversely. The pronotum and elytra are blackish brown with a strong brassy lustre.
^1^) Aehnlich gefärbt ist die nahestehende *PlateumarisWeisei* Duviv. von Irkutsk (Jakowlew), welche Jacobsohn, Horae 26 p. 435 irrthümlich zu den Arten mit behaartem Halsschilde zählt. Das zweite Fühlerglied ist bei ihr stets halb so lang als das dritte, das Halsschild ähnelt dem von *consimilis* ist aber bedeutend schmaler und flacher, die Punktirung der Flügeldecken ist ziemlich dieselbe wie bei *Donaciacuprea*.	^1^) The nearby *PlateumarisWeisei* Duviv. from Irkutsk ([leg.] Jakowlew) is similarly coloured, which Jacobsohn, Horae 26 p. 435
	[= reference to Jakobson [= Jacobson, Jacobsohn] G. G. 1892a: Analytische Übersicht der bekannten Donacia- und Plateumaris-Arten der Alten Welt. Horae Societatis Entomologicae Rossicae 26: 412–437] erroneously aligned with the species with a hairy pronotum. The second antennal segment is here [concerning *P.weisei*] always half as long as the third one, the pronotum resembles that of [*Plateumaris*] *consimilis* but is significantly narrower and flatter, the puncturing of the elytra is rather the same as in *Donaciacuprea* [= *Donaciasemicuprea*, Panzer, 1796].

### ﻿*Plateumarisannularis* Reitter, 1920

Described on page 41. Synonym of *P.roscida*.

**Table:** 

**German**	**English**
***Plateumarisannularis***	***Plateumarisannularis***
Eine Art, die in der Mitte zwischen *discolor* und *sericea* steht, da sie zum Teil Eigenschaften der beiden vereinigt. Die Fühler sind auffallend dünn, von der Form, wie bei *sericea*, aber die Glieder breit gelbrot geringelt. Halsschild ebenso wie bei *sericea*, aber ohne längs der Mitte eingerissene Mittellinie und die Lateralbeulen ganz flach und nicht glänzend, die metallischen Schenkel und Schienen an der Basis fast bis zur Mitte gelbbraun, Pygidium und Apikalsaum des Analsternites rot ; kupferig, erzfarben.	A species that stands in the middle between *discolor* and *sericea*, since it partly combines characteristics of both. The antennae are remarkably thin, the same form as in *sericea*, but the segments are broadly annulated in yellow-red. Pronotum as in *sericea*, but without median line engraved along the centre, and the lateral tubercles quite flat and not shiny, the metallic femora and tibiae from the base almost to the middle yellow-brown, pygidium and apical margin of anal sternite red; coppery, bronze.
L. 8–9 mm.	L[ength] 8 – 9 mm.
Ost Sibirien: Amurgebiet. (Chabarowka, Nikolajewsk, in Col. Koltze.)	East Siberia: Amur region. (Chabarowsk, Nikolajewsk, in col[lection] Koltze.)

### ﻿*Plateumarisassimilis* (Schrank, 1781)

Described as *Lepturaassimilis* on page 156. Synonym of *P.consimilis*.

**Table:** 

**Latin**	**English**
***Lepturaassimilis***	***Lepturaassimilis***
Vergleichbarer Holzkäfer [translation into in German]	Comparable xylobiontic beetle
Leptura nigra; pedibus testaceis ; posticis dentatis; elytris simplicibus.	Black xylobiontic beetle; legs testaceous, hind ones with a tooth; elytra simple.
Mensurae. Longit. a cap. ad an. 3 ½ lin. elytri 2 ^1^/_5_ – Latitudo insecti 1 ^1^/_3_ –	Measurements: *) Length from head to anus 7.7 mm of an elytron 4.8 mm Width of the insect 2.9 mm
Descr. Nigra; elytro singulo striis novem punctatis. Pedes testacei, femora postica dentata.	Descr[iption] Black; the single elytron with nine rows of punctures. Legs testaceous, hind femora with a tooth.

*) Calculated from the unit “Vienna line”: 1 lin. = 2.195 mm. This unit would most likely be used by Schrank, especially in a book about Austrian insects.

### ﻿*Plateumarisbracata* (Scopoli, 1772)

Described as *Prionusbracatus* on page 100.

**Table:** 

**Latin**	**English**
***PRIONUS Bracatus***	***PRIONUS Bracatus***
Diagn. Niger; antennis pedibusque rufis. Femoribus posticis crassis, spina armatis.	Diagn[osis:] Black; antennae and legs red. Hind femora thick, armed with a spine.
In Carniolia.	In Carniola [historical region in West Slovenia].
Elytra lin. 3–4. longa, non truncata, punctata: punctis in lineas ordinatis. Antennae breves.	Elytra *) 6.8 – 9 mm long [calculated from Parisian line], not truncated, punctured: dots arranged in rows. Antennae short.

*) Calculated from the unit “Parisian line”: 1 lin. = 2.2558 mm. This unit would most likely be used by Scopoli.

Because this original description was very short, Weise (1893) published a more detailed redescription on page 49.

**Table:** 

**Latin**	**English**
***P..braccata***	***P..braccata***
Elongata, convexiuscula, supra violaceo-nigra, subtus griseo-vel aureo-sericea; abdomine basi exepta, pedibus antennisque rufo-ferrugineis; prothorace subcordato, viridescens, canalicula media obsoleta, angulis anticis vix prominulis; elytris apice conjunctim-rotundatis, striato-punctatis, interstitiis transversim rugosis. Long. 9–11 mm.	Elongated, slightly convex, purplish black above, below grey or silky golden; abdomen except base, legs and antennae light reddish-rusty brown; the prothorax subcordate, greenish, the central groove inconspicuous, the front angles hardly protruding; the elytra rounded together at the apex, with dotted stripes, the intervals transversely wrinkled. Length 9 – 11 mm.
Mas[culus]: prothoraces parce punctato, nitido, metasterno et segmento primo medio late impresso, 5: o apice emarginato; femoribus posticis dente valido armatis.	Male: prothorax sparsely punctured, shiny, metasternum and the first abdominal segment in the middle broadly impressed, the 5^th^ one at the tip emarginated; the hind femora armed with a strong tooth.
Fem[inae]: prothorace crebre punctato, segmento 5: o apice subtruncato, femoribus posticis obsolete dentatis vel muticis.	Female: prothorax densely punctured, the 5^th^ segment somewhat truncated at the apex, the hind femora with an inconspicuous tooth or blunted.
Var. a. Antennis, pedibus abdomineque nigris.	Var[iation] a. Antennae, legs, and abdomen black.
Var. b. (femina). Supra aenea, thorace chalybaeo-micans. Suffr. Stett. Zeit. 1846. 56.	Var[iation] b. (female). Upper side bronze-coloured, pronotum shiny like steel. [reference:] Suffr[ian] Stett[iner entomologische] Zeit[ung] 1846: 56.
[Reference first description:] Scopoli Annus V. Hist. nat. [p.]100.	
[Synonyms:] *Donacianigra* Fabr[icius]. Ent. Syst. I. 2. 117. – Germ[ar] Neue Schrift. Ges. Halle VI. 31. – Lac[ordaire] Mon. 171. 46. – Redtb [Redtenbacher] Faun[a] A[ustriaca] II. 441. – Seidl[itz] F[auna] balt[ica] 508.	
*D.palustris* Herbst. Füessl. Arch. V. 100. – Panz[er] Ent. Germ. 217. 13; Faun. Germ. 29. 10.	

### ﻿*Plateumariscaucasica* Zaytsev, 1930

Described on page 111. Synonym of *P.sericea*.

**Table:** 

**Latin**	**English**
***Plateumariscaucasica***	***Plateumariscaucasica***
♂♀. Species inter *sericea* L. et *discolor* Panz. intermedia, sed illius manifeste affinior.	♂♀. Intermediate species between *sericea* L. and *discolor* Panz., but clearly closer to the former.
Ab ambobus differt pedum colore: femoribus in triente basali, tibiarum parte ¾ longitudinis (nonnunquam usque ad apicem), tarsorum articulis omnibus (ultimo saepe excepto), segmentorum abdominalium summo margine, pygidio rufo-ferrugineis.	It differs from both by the colour of the legs: the femora at the basal third, the tibiae at 3/4 of the length (sometimes up to the apex), all tarsomeres (often except the last one), the very margin of the abdominal segments and the pygidium are reddish-rubiginous.
Praeterea a *P.sericea* discrepat corporis forma robustiore, subtus brevius pubescente, antennis gracilioribus atque longioribus, rubro-castaneis, articulis solum apice vel nihilo obscuratis, pronoto fortius ruguloso (fere ut in *P.dicolor* [sic!]) angulis anticis haud prominulis; ceterum cum *P.sericea* congruens.	Further it differs from *P.sericea* by a more sturdy, ventrally shorter and densely pubescent physique, more slender and longer testaceous antennae with antennomeres darkened only at the apex or nowhere, a more rugose pronotum (almost like *P.dicolor* [sic!]) with not protruding anterior angles; otherwise consistent with *P.sericea*.
A. *P.discolor* magis adeo distat antennis gracilibus, articulo quarto quam secundo duplo longiore etc. Superficiei colore variat viride vel aurichalceo.	Many more differences exist compared with *P.discolor* by more slender antennae, the fourth antennomere which is twice as long as the second one etc. The surface colour varies between green and brassy.
Long. 7–8,5 mm.	Length 7–8.5 mm.
Hab. ‘Ciscaucasia: Stavropol (IV. 1905, Maljuzhenko, 4 specimina), Daghestan: Chasav-jurt (E. Koenig, 5 sp.). – Coll. Musei Georgici.	Dis[tribution] ‘Ciscaucasia: Stavropol (IV. 1905, Maljuzhenko, 4 specimens), Daghestan: Chasav-jurt (E. Koenig, 5 sp.). [stored in the] Coll[ection] of the Georgian Museum.
*P.annularis* Reitt. (e prov. Amurensis) verisimiliter maxime affinis, species nostra tamen secundum auctoris descriptionem corpore minore, tibiis amplius rufo-coloratis diversa esse videtur.	Probably very close to *P.annularis* Reitt[er] (from the Amur Prov[ince]), but it seems that our species differs according to the author’s description by a smaller body and more extensively red-coloured tibiae.
**Russian**	**English**
Хотя указанных сейчас отличий от восточно-сибирского вида и недостаточно для признания видовой самостоятелъности за нашей формой, но разорванностъ ареалов обитания, лаконичностъ описания у Reitter’a и отсутствие для сравнения амурских представителей этого вида дают основания пока считатъ их различными видами.	Although the differences now indicated from the East Siberian species are not enough to regard this form as an independent species, the fragmentation of the habitats, the brevity of Reitter’s description and the lack of comparison with specimens from the Amur give reasons to consider it as a different species for the time being.

### ﻿*Plateumarisconsimilis* (Schrank, 1781)

Described as *Lepturaconsimilis* on page 155.

**Table:** 

**Latin**	**English**
***Lepturaconsimilis***	***Lepturaconsimilis***
Aehnlicher Holzkäfer [German vernacular name]	Similar xylobiontic beetle
Leptura aenea; antennis pedibusque testaceis, femoribus posticis dentatis; elytris simplicibus.	Bronze-coloured xylobiontic beetle; antennae and legs testaceous, hind femora with a tooth; elytra simple.
Mensurae. Longit. a cap. ad an. 4 lin. elytri 2 ½ – Latitudo elytri ^2^/_3_ –	Measurements: *) Length from head to anus 8.78 mm of an elytron 5.48 mm Width of an elytron 1.46 mm
Descr. Nigro-aurea, subtus obscurior. Antennae fusco – testaceae, pedes rubro –testacei, postici femoribus dentatis.	Description: black-golden, underside darker. Antennae brown-testaceous, legs red-testaceous, hind ones with toothed femora.
Elytra non lacunosa, striis singular decem punctatis exarata.	Elytra without impressions, each one furrowed with ten rows of punctures.

*) Calculated from the unit “Vienna line”: 1 lin. = 2.195 mm. This measure would most likely be used by Schrank, especially in a book about Austrian insects.

### ﻿*Plateumarisdiscolor* (Panzer, 1795)

Described as *Donaciadiscolor* on page 216. Synonym of *P.sericea*.

**Table:** 

**Latin**	**English**
***Donaciadiscolor***	***Donaciadiscolor***
obscure aenea, elytris cupreis crenato striatis, femoribus posticis dentatis. Habitat in Caltha palustri primo vere. (Variat elytris aeneo nitidulis et obscure cupreis. Elytra linearia, obtusa nec apice attenuata. Femora postica in utroque sexu dentata.)	dark bronze, the copper-coloured elytra with notched grooves, the hind femora dentate. Dwells in the marsh marigold in early spring. (Varies with shiny bronze and dark copper-coloured elytra. The elytra straight, blunt and not narrowed towards the tip. The posterior femora in both sexes dentate.)

### ﻿*fairmairi*: Plateumarisbracatavar.fairmairi (LeGrand, 1861)

Described as variation of *Donacianigra* (synonym of *Plateumarisbracata*) on page 265.

**Table:** 

**French**	**English**
**Donacianigravar.fairmairi**	**Donacianigravar.fairmairi**
D[onacia] nigra Fab[ricius]	D[onacia] nigra Fab[ricius]
Dans les fossés du château de Regnault. R.R. [Département de l’Aube]	In the moats of the castle of Regnault. R.R. [Department Aube]
– Var. A. Lacordaire. D. Fairmairi Nobis.	Var. A. Lacordaire. D. Fairmairi Nobis.
Avec le précédent, trouvée une seule fois.	With the previous one [= *Donacianigra*, synonymous with *Plateumarisbracata*], found only once.
Cette variété, mentionnèe par M. Lacordaire, comme ayant été trouvée par Dejean, en Dalmatie, se distingue par son abdomen, ses pieds et ses antennes entiérement noirs.	This variety, which was mentioned by M. Lacordaire as having been found by Dejean in Dalmatia, is distinguished by its entirely black abdomen, legs and antennae.

### ﻿*Plateumarisintermedia* Apfelbeck, 1912

Described on page 239. Synonym of *P.sericea*.

**Table:** 

**Latin**	**English**
***Plateumarisintermedia***	***Plateumarisintermedia***
*D.sericeae* L. simillima, antennis crassioribus, earum articulis rubro-variegatis; prothorace longiore, angulis anticis rotundatis, tuberculis lateralibus obtusis; elytris longioribus, planioribus, antrorsum densius punctatis; a *D.discolore* Panz. antennarum articulo secundo et tertio longiore (ut in *D.sericea* L.); prothorace longiore et planiore, supra magis depresso, antrorsum dilatato, ad marginem apicalem latissimo, sericeo, haud vel vix rugoso, subtiliter, aequaliter et confertissime punctato; elytris longioribus et planioribus, subnitidis, confertim punctatis distinguenda.	Very similar to *D*[*onacia*] *sericea* L., antennae thicker, antennomeres red-variegated; pronotum more elongated, anterior corners rounded, lateral tubercles blunt; elytra more elongated, more flattened, in basal part more densely punctate; it can be distinguished from *D*[*onacia*] *discolor* Panz. by a longer second and third antennal segment (as in *D.sericea* L.); the pronotum is longer and flatter, upper side more depressed, widening forward, at the apical margin very broad, silky, not or hardly wrinkled, punctured faintly, evenly and densely; elytra longer and more flattened, rather shiny, densely punctured.
Bosnia c. (Jezero) et occ. (Livno).	In central (Jezero) and western Bosnia (Livno).
[Then the very same description is printed in Serbian language in Cyrillic letters. Only the location data are slightly more detailed]:	
**Serbian**	**English**
Средња и западна Босна: Језеро код Јаjца, Басташи код Ливна (Reiser) на *Cladiummariscus*-y.	Central and western Bosnia: Jezero near Jajca, Bastaši near Livno ([leg.?] Reiser) on *Cladiummariscus*.

### ﻿*Plateumarislacordairii* (Perris, 1864)

Described as *Donacialacordairii* on page 300. Synonym of *P.sericea*.

**Table:** 

**Latin**	**English**
***DonaciaLacordairii***	***DonaciaLacordairii***
Supra viridi-ænea, nitida, juxta suturam subviolacea; subtus plumbeo-ænea, opaca, argenteo-sericea;	Green-bronze coloured above, shiny, somewhat violet near the seam; below lead-grey-bronze, dull, silvery-silky;
capite fortiter, densissime et rugose punctato; antennis nigris, articulis quinque ultimis basi rubris;	the head with strong, very dense and wrinkled punctures; the antennae black, the last five segments red at the base;
prothorace subdeplanato, tenuiter, densissime et fere reticulatim ruguloso, canaliculato, basi angustiore et transversim foveolato; angulis anticis prominentibus;	the prothorax somewhat flattened, delicately, very densely wrinkled, almost like a net, furrowed, narrower at the base and with dimples across; the front corners protruding;
elytris sat convexis præsertim postice, apice truncato-rotundatis, fortiter striato-punctatis; interstitiis striarum transversim strigosis;	the elytra are conspicuously arched, especially at the back, truncated-rounded at the apex, with strong dotted striae; the spaces between the striae striped across;
pedibus crassis, brevibus, femoribus inflatis; posticis fortiter et acute unidentatis.	legs thick, short, femora swollen; the rear ones with a strong and sharp tooth.
Long. 7 mill.	Length 7 mill[imeters]
**French**	**English**
Antennes noires, leurs cinq derniers articles rouges à la base; 3^e^ article une fois et demie aussi long que le 2^e^, plus court que le 4^e^.	Antennae black, their last five segments red at the base; 3^rd^ antennomere one and a half times as long as 2^nd^, shorter than 4^th^.
Tête presque plane, recouverte d’une pubescence soyeuse, argentée; très densément et comme rugueusement ponctuée; front un peu convexe, longitudinalement sillonné.	Head almost flat, covered with silky, silvery pubescence; very densely and roughly punctate; slightly convex forehead, furrowed longitudinally.
Prothorax plus long que large, plus étroit à la base qu’au sommet, peu convexe, canaliculé au milieu, marqué à la base d’une fossette transversale et triangulaire; dilaté sur le côtés au-dessous des angles antérieurs qui sont saillants en forme de dent obtuse et un peu rejetée en arrière; tout couvert de petites rides ou d’une sorte de réticulation très confuse et très serrée. Écusson subtriangulaire, très finement soyeux.	Prothorax longer than wide, narrower at the base than at the top, not very convex, channelled in the middle, marked at the base with a transverse and triangular dimple; dilated on the sides below the anterior angles which are projecting in the form of an obtuse tooth and a bit reflected; all covered with little wrinkles or some sort of very confused, very tight reticulation. Subtriangular dorsal disk, very finely silky.
Élytres à vagues reflets violacés le long de la suture et principalement autour de l’écusson; marquées d’une dépression transversale au tiers antérieur et d’une autre peu visible un peu au-delà du milieu; assez convexes, surtout postérieurement; subtronquéesà l’extrémité ; fortement striées-ponctuées ; points der stries très rapprochés ; intervalles transversalement ridés.	Elytra with vague purplish reflections along the suture and mainly around the dorsal disk; marked with a transverse depression in the anterior third and another inconspicuous one a little behind the middle; quite convex, especially posteriorly; truncated at the apex; strongly striate-punctate; stitches of very close striae; transversely wrinkled interstices.
Dessous du corps d’un noirâtre un peu bronzé, revêtu d’une pubescence soyeuse, argentée, très serrée.	Underside of the body a little tanned blackish, covered with a silky, silvery, very dense pubescence.
Pattes de la même couleur, courtes épaisses, cuisses très renflées, les postéreures munies d’une forte dent triangulaire.	Legs of the same colour, short thick, very swollen thighs, the posterior ones provided with a strong triangular tooth.
Elle se place dans la mème division que la *D.sericea* L. et elle a de grands rapports avec elle. Elle en diffère néanmoins par de caractères bien tranchés. Les tubercules placés derrière les angles antérieurs du prothorax sont moins saillants ; à partir de ces tubercules les côtes sont un peu arqués en dedans dans la *sericea* et la base finit par avoir ls même largeur que le sommet; dans la *Lacordairii* le prothorax se rétrécit au contraire insensiblement jusqu’à la base en s’arrondissant très légèrement ; il est en outre plus court. Les ponts des stries des élytres sont plus rapprochés, et par-dessous tout les pattes sont plus courtes, plus épaisses et les cuisses sensiblement plus renflées.	It is placed in the same division as *D.sericea* L. to which it is closely related. However, it differs from it by well-defined characters. The tubercles placed behind the anterior angles of the prothorax are less protruding; from these tubercles the ridges are arched inwards in *sericea*, and the base ends up with the same width as the anterior part; in *Lacordairii* the prothorax narrows, on the contrary, imperceptibly to the base, rounding out very slightly; it is also shorter. The bridges of the elytral striae are closer together, and below all the legs are shorter and thicker, and the thighs noticeably more swollen.
J’ai pris cette espèce en Espagne, aux bords d’un ruisseau sur la route de la Granja à San Rafaël. Je la dédie à mon illustre ami M. Lacordaire, comme témoignage d’affectueuse admiration pour son caractère et ses travaux.	I caught this species in Spain, on the banks of a stream at the road from La Granja to San Rafaël. I dedicate it to my illustrious friend M. Lacordaire, as a testimony of affectionate admiration for his character and his work.

### ﻿*Plateumarismongolica* (Semenov, 1895)

Described as Donacia (subgenus Plateumaris) mongolica on page 267. Synonym of *P.weisei*.

**Table:** 

**Latin**	**English**
**Donacia (Plateumaris) mongolica**	**Donacia (Plateumaris) mongolica**
♂. Minor, sat debilis, modice convexa, capite, prothorace corporeque subtus obscure viridi-aeneis, nitidis, elytris obscure cupreis, opacis, suturae margine infero postice late patente aeneo-nigro, nitido, pedibus, ore antennisque dilute testaceis, his ad apicem leviter infuscatis, segmentorum abdominalium margine postico plus minusve rufescenti.	♂. Smaller, rather weak, medium convex, head, prothorax and underside of the body dark green-ore-coloured, shiny, elytra dark cupreous, elytra dark copper-coloured, gloomy, the lower margin of the suture, which is wide open at the back, is ore-black, shiny, legs, mouth and antennae are wanly clay-coloured, these are slightly browned towards the apex, the posterior margin of the abdominal segments is more or less reddish.
Antennis dimidium corpus saltem sesqui superantibus, articulo 2° tertio plus quam sesqui breviore, 4° tertium fere 11/3 superante. Capite confertim punctulato, tenuiter pubescenti, vertice utrinque oculos versus praeterea tenuissime ruguloso, sulco longitudinali medio profunde impresso postice abrupte abbreviato, sulcis juxtaorbitalibus nullis; oculis parvis extrorsum valde prominentibus ; temporibus pone oculos breviter inflatis, hos nonnihil amplectentibus, deinde fortiter constrictis: genis diametro oculorum fere aequilongis. Mandibulis validiusculis labrum multo superantibus.	Antennae at least half longer than half the body [antennae ¾ total body length], the 2^nd^ segment more than half shorter than the third one, the 4^th^ one ca. 11/3 times longer than the third one. The head is densely punctured, finely hairy, the vertex in addition very finely wrinkled on both sides towards the eyes, the deeply depressed central longitudinal furrow abruptly shortened behind, without furrows next to the eye sockets; the small eyes protruding distinctly outwards; the temples shortly inflated behind the eyes, enclosing them a little, then strongly contracted: the cheeks about the same length as the diameter of the eyes. Mandibles somewhat strong, much longer than the labrum.
Prothorace subquadrato latitudine distincte longiore, lateribus mox pone angulos anticos denticuliformes breviterque extrorsum prominulos leviter tumido et ibi summam latitudinem attingente, dein basin versus sensim vix distincte subangustato, ante angulos posticos non sinuato, his (aspectu desuper) extrorsum paulo prominulis, puncto setigero notatis; apice recte truncato, basi utrinque ad angulos posticos sat fortiter obliquata; disco parum convexo, nitidulo, haud crebre (medio fere disperse) subtiliter punctato, minutissime parce pubescenti, utrinque ad angulum anticum subimpresso subtilissimeque vix distincte ruguloso, paulo ante medium trinque tuberculo indeterminato nitido signato, linea media obsoleta vel omnino obliterata solum ante basin profunde foveatim impressa.	The almost square pronotum is distinctly longer than wide, slightly swollen on the sides just behind the tooth-shaped and slightly outwards protruding anterior corners, and reaches its greatest width there, then noticeably and vaguely slightly narrower towards the base, not sinuated in front of the posterior corners, these (viewed from above) slightly protruding outwards, marked by a bristle-bearing point; the end truncated straight across, the base on both sides very sharply bevelled towards the rear corners; the disc is slightly convex, slightly shiny, not densely (almost scattered in the middle) finely punctured, with extremely tiny and sparse hairs, slightly dented on both sides towards the front angle with extremely fine and rather indistinct wrinkles, slightly in front of the middle on both sides marked with an indistinct shiny tubercle, the inconspicuous or completely obliterated median line deeply depressed and excavated only in front of the base.
Elytris prothoracis basi duplo latioribus, summa latitudine circiter 12/3 longioribus, pone medium levissime ampliatis, deinde ad apicem sat abrupte angustatis, apice singulatim simpliciter angustato-rotundatis, dorso convexiusculis, impressionibus prorsus destitutis, tenuius striato-punctatis, interstitiis fere planis vel vix convexiusculis, confertim alutaceis, subopacis, 1° (juxtasuturali) inde ab apice striae juxtascutellaris elongatae valde convexo, calloso-elevato, nitidiusculo, 9° pone humerum plicam crassiusculam efficiente; sutura posterius fortiter replicata, margine infero laevi, nitido; humeris rectis modice obtusis.	The elytra twice as wide as the base of the pronotum, ca. 11/3 times as long as the maximum width, very slightly widened behind the middle, then narrowed rather abruptly towards the end, at the end each one individually simply narrowed-rounded, slightly arched on the back, completely without impressions, with rather narrow punctured stripes, the intervals almost flat or hardly slightly arched, densely leather-like, rather dark, the 1^st^ one (next to the seam) very arched from the end of the extended stripe next to the scutellum, bulging-raised, slightly shiny, the 9^th^ one forming a podgy fold behind the shoulder; the seam is well folded back at the rear, the lower edge is smooth and shiny; the straight shoulders moderately blunted.
Subtus corpore toto nec non pygidio haud dense longiusque subsericeo-cano-pubescentibus. Abdomine confertissime punctulato; segmento basali duobus sequentibus unitis vix longiore, simplici: neque impresso neque tuberculato; segmento anali apice late et fere recte truncato, haud impresso.	Below, the whole body and also the pygidium are not densely covered with quite long, silky grey hairs. The abdomen very densely punctured; the basal segment scarcely longer than the following two together, simple: neither indented nor bulging; at the end the anal segment is truncated broadly and almost straight, not indented.
Pedibus haud longis, validiusculis; femoribus crassis omnibus ad apicem valde inflatis, posticis marginem apicalem segmenti penultimi abdominalis haud vel vix superantibus, infra ante apicem dente valido late triangulari, nonnihil retrorsum directo munitis; tibiis omnibus simplicibus, integris, ad apicem leniter sensimque dilatatis, anticis apice extus breviter subproductis; tarsis articulo penultimo lobis modice elongatis.	Legs not long, quite strong; the thick femora all very expanded towards the end, the rear ones not or hardly exceeding the rear edge of the penultimate abdominal segment, reinforced below before the end with a strong, broad, triangular tooth that points a little backwards; all tibiae simple, complete, slightly and noticeably widened towards the end, the anterior ones shortly and slightly protruding outwards at the end; the tarsi on the penultimate segment moderately lengthened by lobes.
Long. 6½, lat. ad humer. 23/5 mm.	Leng[th] 6½, wid[th] at the shoul[ders] 23/5 mm.
Mongolia septentr.: vallis fluvii Borcha, orientem versus ab Urga (B. Kaschkarow. 6. VII. 1894). – Specimen unicum ♂ (coll. P. a Semenow). Mera subgeneris *Plateumaris* C. G. Thoms. ^16^) species.	North[ern] Mongolia: valley of the river Borcha, from Urga towards the east (B. Kaschkarow. 6. VII. 1894). – A single specimen ♂ ([in] coll. P. of Semenov). A real species of the subgenusPlateumaris C. G. Thoms. ^16^).
A *D.n.abdominali* Oliv. (*affini* Kunze) cui proxima, differt imprimis statura debiliore, antennis gracilioribus articulis omnibus magis elongatis, 4° praecedente distincte longiore, prothorace angustiore lateribus pone angulos anticos extrorsum fere denticulatim prominulos fortius inflato, disco parcius punctato, magis lucido, elytris ad apicem magis abrupte angustatis, dorso paulo tenuius striato-punctatis, interstitio primo inde ab apice striae juxtascutellaris calloso-elevato, 9° pone humerum plicam abbreviatam formante, suturae margine infero posterius magis patente, abdominis segmento basali in ♂ simplici brevique, metasterno haud impresso quoque, femoribus posticis in eodem sexu dente validiore et obtusiore armatis, tibiis gracilioribus, etc.	From *Don.abdominalis* Oliv. (*affinis* Kunze) *), to which it is closest, it differs above all by the weaker stature, by the more delicate antennae with consistently longer antennomeres, the 4^th^ one clearly longer than the previous one, by the narrower pronotum with sides more swollen behind the anterior corners, almost dentiformly protruding outwards, by the more sparsely dotted, lighter disc, by the elytra more abruptly narrowed towards the end, with a little narrower dot-stripes on the back, by the bulging-raised first interval from the end of the stripe next to the scutellum, by the 9^th^ one forming a shortened fold behind the shoulder, by the lower edge of the seam more gaping further back, by the simple and short basal abdominal segment in the ♂, by the metasternum, which is also not impressed, by the hind femora armed with a stronger and more blunted tooth in the same sex, by the more delicate tibiae, etc. *): *Donaciaabdominalis* Olivier is now synonym with *Plateumarisbracata* and not with *P.affinis* Kunze.
A *D.n.consimili* Schrank, cui affinis quoque, discedit praesertim iisdem notis atque a *D.abdominali*. A *Don.rustica* Kunze praeterea colore antennarum pedumque discrepat.	Above all, from *Don.consimilis* Schrank, to which it is also closely related, it differs by the same characters as from *D.abdominalis*. Furthermore, it also differs in the colour of the antennae and legs from *Don.rustica* Kunze.
A *D.n.Weisei* Duviv. ^17^) differt imprimis prothorace disco nitido haud confertim punctato, angulis anticis extrorsum denticulatim prominulis, elytris opacis interstitio primo pone striam juxtascutellarem fortiter calloso-elevato, 9° pone humerum breviter plicato.–Facile tamen fieri potest, ut *D.mongolica* m. nil nisi maris *Donaciae Weisei* (mihi prorsus ignotae) mera sit aberratio.	From *D.n.Weisei* Duviv. ^17^) it differs above all by the shiny and not densely dotted pronotal disc, with tooth-shaped and outwards protruding front corners, by dark elytra with the first interval behind the stripe next to the scutellum, which is very bulging and raised, and the 9^th^ one behind the shoulder that is briefly folded up. –Nevertheless, it is easily possible that m[y] *D.mongolica* is just a simple aberration of the male of *DonaciaWeisei* (which is totally unknown to me).
^16^) Genus *Plateumaris* (C. G. Thoms.) Weise, Jacobs. ad gradum subgeneris reducendum esse censeo; nam nonnullae species orientali-asiaticae, imprimis *Plateumarisexcisipennis* Jacobs. (Horae Soc. Ent. Ross., XXVIII, 1894, p. 241), cujus in descriptione auctor sexum speciminis originalis unici indicare verisimiliter invitus neglexit, transitum nimis manifestum ad genus *Donacia* F. praebere videntur. Ceterum praesumo *Donaciamexcisipennem* (Jacobs.) subgenus proprium constituere.	^16^) I think that the genus *Plateumaris* (C. G. Thoms.) Weise, Jacobs. should be relegated to the category of a subgenus; because a number of East Asian species, above all *Plateumarisexcisipennis* Jacobs. (Horae Soc. Ent. Ross., XXVIII, 1894, p. 241), where the author probably reluctantly failed to indicate the sex of the only original specimen in its description, seems to provide a very clear transition to the genus *Donacia* F. Incidentally, I suspect that *Donaciaexcisipennis* (Jacobs.) establishes its own subspecies.
^17^) Duvivier: Ann. Soc. Ent. Belg., XXIX, 1885, Bull., p. CXVI. – Jacobson: Horae Soc. Ent. Ross., XXVI, 1892, p. 435.	^17^) Duvivier: Ann. Soc. Ent. Belg., XXIX, 1885, Bull., p. CXVI. – Jacobson: Horae Soc. Ent. Ross., XXVI, 1892, p. 435.

### ﻿*Plateumarisnigra* (Fabricius, 1792)

Described as *Donacianigra* on page 117. Synonym of *P.bracata*.

**Table:** 

**Latin**	**English**
***Donacianigra***	***Donacianigra***
6. D.nigra elytris substriatis, abdomine pedibusque rufis.	6. Black D[onacia] with somewhat striped elytra, abdomen, and legs red.
Habitat in Germaniae aquis Dom. Smidt.	Inhabits waters of Germany [according to] Mr. Smidt.
Statura & summa affinitas D. Festucae. Antennae nigrae: primo articulo rufo. Caput & thorax nigra, immaculata, nitidula. Elytra minus striata minusque depressa. Adomen rufum. Pedes rufi femoribus posticis in altero sexu simplicibus in altero dentatis.	Stature and next of kin to D. Festucae *). Antennae black: first segment red. Head & chest black, spotless, shiny. Elytra less striped and less depressed. Abdomen red. Legs red, hind femora simple in one sex, dentate in the other.

*) *D.festucae* (Fabricius, 1792): 116: synonym of *P.sericea*

### ﻿*Plateumarisobsoleta* Jacobson, 1894

Described on page 243. Probable synonym of *P.shirahatai*.

**Table:** 

**Latin**	**English**
***Plateumarisobsoleta***	***Plateumarisobsoleta***
E divisione prima opusculi mei (Horae Soc. Ent. Ross. XXVI, p. 433), cujus inter species *discolorem* Pz. et *sericeam* L. ponenda.	From the first section of my work (Horae Soc. Ent. Ross. XXVI, p. 433), to be placed between its species *discolor* Pz. and *sericea* L.
Convexiuscula, aurichalcea; corpore subtus, capite, scutello, pedibus antennisque argenteo-tomentosis. Cuput [sic! a typing error, it should be: Caput] densissime punctatum, linea media impressa frontis tenui, profunda.	Slightly convex, brass coloured; underside of the body, the head, the scutellum, legs and antennae silvery tomentose. The head extremely densely punctured, the impressed narrow median line in the head deep.
Antennae dimidio corpori longitudine aequales, sed multo magis tenues quam in *P..discolore*, articulo primo crasso, ceteris subtilibus, articulo 2° sesqui breviore quam tertius, 4° hoc sesqui longiore, ceteris subaequali; articulorum 2^i^–11^i^ basibus rufo-ferrugineis.	Antennae in length equal to half the body, but much thinner than in *Pl.discolor*, the first segment thick, the others fine, the 2^nd^ segment is half as short as the third one, the 4^th^ one by half longer than this one, the rest almost the same; the basis of the 2^nd^–11^th^ segment is reddish-rusty brown.
Prothorax latitudine multo longior, disco subplano, lateribus subparallelis; tuberculo laterali minime convexo, parum determinato; angulis omnino non prominulis, rectis; linea longitudinali disci vix distincta; disco subtilissime remote punctato, inordinate subtiliter et dense ruguloso, nudo, sericeo-opaco (basi nitida excepta).	Prothorax much longer than wide, the disc nearly flat, the sides nearly parallel; lateral tubercles minimally convex, not very pronounced; the angles not a bit protruding, straight; the longitudinal line of the disc scarcely pronounced; the disk very finely widely punctured, irregularly, finely and densely wrinkled, hairless, silky dusky (except the shiny base).
Elytra lateribus subparallelis, a triente ultimo ad apicem rotundato-truncatum angustata, dorso juxta suturam leviter biimpressa, haud fortiter striato-punctata, inter puncta rugulis transversis haud altis ornata, nitida.	The elytra with almost parallel lateral sides, narrowed from the last third to the rounded, truncated apex, slightly impressed twice on the back next to the suture, not strongly striped-punctured, decorated with non-raised transverse wrinkles between the dots, shiny.
Abdomen segmento primo medio plano; segmento anali apice rotundato. Pygidium apice rotundatum.	First segment of abdomen flat in the centre; anal segment apically rounded. The pygidium apically rounded.
Pedes corpori concolores, articulationibus omnibus unguiculisque rufo-ferrugineis; femoribus posterioribus omnino inermibus, subtus solum obtuse angulatis.	The legs with the same colour as the body, all joints and the claws reddish-rusty brown; the hind femora completely unarmed, only bluntly angled below.
Long. 7 mm.	Length 7 mm.
Sibiria orientalis: sinus Possiet. Specimen unicum (♂).	East Siberia: Bay of Posyet. Single specimen (♂). [In fact, this is a female specimen. The protruding ovipositor was mistaken for the aedeagus (Geiser 2023: figs 14b, 15b).]

### ﻿*orientalis*: *Plateumarisconsimilisorientalis* Shavrov, 1948

Described as subspecies on page 49. Synonym of *P.weisei*.

**Table:** 

**Latin**	**English**
***Plateumarisconsimilisorientalis***	***Plateumarisconsimilisorientalis***
Corpore supra splendidiore, rugulis striarum interstitiorum elytrarum humilioribus, dente femorum posticorum minore vel obsoleto.	Body shinier above, the wrinkles between the grooves of the elytra lower, tooth on the hind femora smaller or extinct.
**Russian**	**English**
Размеры тел и соотношение длин и ширин всех частей те же, что и у типичной формы, но тело значителъно более блестящее, благодаря менее грубой скулъитуре надкрылий.	The dimensions of the body and the ratio of the lengths and widths of all parts are the same as in the typical form, but the body is much more lustrous due to the less coarse sculpture of the elytra.
Точечные ряды надкрылий менее углублены, поперечные морщины промежутков, как основные так и мелъчайшие, выражены менее резко и более сглажены. Пунктировка в основаниях надкрылий более тонкая и менее спутанная. Переднеспика с более резким перехватом за боковыми бугорками.	The dotted grooves of the elytra are less deepened, the transverse wrinkles of the intervals, both the basal and the tiny ones, are less pronounced and smoother. The punctuation at the basis of the elytra finer and less confused. The pronotum with a sharper recess behind the lateral tubercles.
Задние бедра ♀без зубца или с незначителъным бугорком на его месте. Верхняя сторона бронзово – медная с зеленоватым оттенком. Четыре последних брюшных стернита, усики и ноги – краснобурые. Волоски нижней стороны и конечностей золотистые или желтоватые.	Hind femora of ♀without tooth or with a slight tubercle in its place. The upper side is bronze-copper with a greenish tint. The last four ventral sternites, antennae, and legs are reddish brown. Underside the hairs and limbs are golden or yellowish.
Длина 8 мм, такая же как у типичной формы.	Length 8 mm, like that of the typical form.
Владивосток, Седанка 19.VI.37 3 ♀, Н. Н.Филиппов.	Vladivostok, Sedanka 19.VI.37 3 ♀, N. N. Filippov.
*Plateumarisconsimilis* Schrank в прошлом столетии считался видом, распространенным по всей средней и южной Европе и, кроме того, в Сибири (Якобсон, 1892) и в Яапонии (Jacoby, 1885) посколъку в литературе были указания для Иркутска (Солъский, окодо 1870 г.) Урала (Редикорцев, 1908) и Японии (Jacoby, 1885) [originally written: Jacobi], относящиеся к виду *Plateumarisdiscolor* Hoppe (= *P.consimilis* Schrank). Других указаний не были, в промежуточных местностях вид тоже нигде наиден не был. Clavareau (1913) и Reitter (1920) давали без измененй те же сведения, что и Якобсон.	In the last century *Plateumarisconsimilis* Schrank was considered as a species distributed throughout central and southern Europe and furthermore in Siberia (Jakobson, 1892) and Japan (Jacoby, 1885), since there were indications in the literature for Irkutsk (Solsky, about 1870), Ural (Redikortsev, 1908) and Japan (Jacoby, 1885) belonging to the species *Plateumarisdiscolor* Hoppe (= *P.consimilis* Schrank). There were no other indications, and the species was not found anywhere in localities in between either. Clavareau (1913) and Reitter (1920) give the same unmodified information as Jacobson.
По категорическому утверждению Колосова (1930)’, *P.consimilis* Schrank есть чисто западноевропейский вид, восточная граница которого (по материалам его коллекции) проходит по территории Польши, а указания на более восточные местонахождения не верны и относятся к другим видам.	According to the categorical assertion of Kolossow (1930), *P.consimilis* Schrank is a purely Western European species, whose eastern boundary passes through the territory of Poland (according to the materials of his collection), while records from more eastern localities are not correct and refer to other species.
Теперь же нахождение данного вида под Владивостоком вносит полную ясность и подтверждает правильность старых указаний для Иркутска и Японии или во всяком случае очень большую вероятность их.	But now the occurrence of this species near Vladivostok brings complete clarity and confirms the correctness of the old records from Irkutsk and Japan, or at least a very high probability.
Вместе с тем мы имеем теперь сборы P.consimilis Schrank из Черниговской и Полтавской областей и можем считать его видом не только западно-европейским. Указание для Урала (Редикорцев), сделанное по литературным источникам середины прошлого столетия, можно считать лишь вероятным.	At the same time, we now have collections of *P.consimilis* Schrank from the Chernigov and Poltava regions [both sites are in the Ukraine], so we can consider it as a species not only of Western Europe. The indication for the Ural (Redikortsy), made according to the literary sources from the middle of the last century, can only be considered as probable.
Наличие *P.consimilis* Schrank на Дальнем Востоке именно в форме особой расы при условии такого большого территориального разрыва предста-вляется вполне естественным. Структурные различия от западно формы в виде большего блеска и меньшей морщинистости надкрылий, дерехвата переднеспинки и более светлого пвета ноги усиков совершенно аналогичны таким же признакам у дальневосточных подвидов других видов донаций, нак, например, *D.clavipesglabrata* Solsky, *D.obscurasplendens* Jacobs. и *D.thalassinarufovariegata* Jacobs.	The presence of *P.consimilis* Schrank as a separate race in the Far East seems quite natural given such a large territorial distance. Structural differences from the western form by stronger shine and less wrinkling of the elytra, the more straight-lined pronotum, and a lighter coloration of the antennae are completely analogous to the same characters in the Far Eastern subspecies of other *Donacia* species, like, for example, *D.clavipesglabrata* Solsky, *D.obscurasplendens* Jacobs. and *D.thalassinarufovariegata* Jacobs.
Пробел между ареалами западной и восточной форм, возможно, будет ваполнен при более подробном изучении фауны СССР, и мы, быть может, получим довольно непрерывное распространение вида с запада на восток, но это только подтвердит наличие довольно типичного восточного подвида *Plateumarisconsimilisorientalis*.	The gap between the distribution areas of the western and eastern form will probably be filled by a more detailed study of the fauna of the USSR, and we may have a fairly continuous distribution of the species from west to east, but this will only confirm the presence of a rather typical eastern subspecies *Plateumarisconsimilisorientalis*.

### ﻿*Plateumarispallipes* (Kunze, 1818)

Described as *Donaciapallipes* on page 35. Synonym of *P.rustica*.

**Table:** 

**Latin**	**English**
***Donaciapallipes***	***Donaciapallipes***
*D.pallipes*: thorace subquadrato, planiusculo, subtiliter impresso, punctulato, angulis anticis truncatis, lateribus subsinuato; elytris depressiusculis, rugulosis, punctis striarum profundis, striisque approximatis, apice rotundatis, pedibus pallescentibus.	*D.pallipes*: the breast somewhat quadrangular, fairly flat, slightly impressed, finely punctured, with truncated front corners, lightly sinuate on the sides; the elytra a little flattened, wrinkled, with deep points in neatly lines, rounded at the apex, the legs paler.

### ﻿*picipes*: Plateumarisrusticavar.picipes Weise, 1898

Described as variation of *Plateumarisrustica* on page 180. Synonym of *P.rustica*.

**Table:** 

**Latin**	**English**
**Plateumarisrusticavar.picipes**	**Plateumarisrusticavar.picipes**
Pedibus piceis vel nigris, geniculis interdum obscure ferrugineis.	Legs pitch-brown or black, knees sometimes dark rubiginous.
**German**	**English**
Von rustica habe ich bis jetzt nur ein Stück aus Krain von Stussiner erhalten, welches auf dem Rücken angedunkelte Schenke besitzt, bei den übrigen sind die Beine einfarbig rost-roth. Zwischen normal gefärbten Exemplaren fing Herr A. Fiori in der Umgegend von Modena (Emilia: Jala, März 1894) nun auch abweichende Stücke, von denen er mir ein oberseits schwarzes, auf dem Halsschilde bläulich schimmerndes M und 2 W schickte. Bei ihnen sind die Beine pechschwarz’, in den Gelenken dunkel rostroth, oder einfarbig schwarz. Es ist möglich, dass diese Varietät auch noch in Deutschland aufgefunden wird.	Until now I have received only one specimen of rustica from Stussiner from Carniola, with femora darkened on their upper side, all others have legs which are uniformly rufous. In the vicinity of Modena (Emilia: Jala, March 1894) Mr. A. Fiori now also caught differing specimens between normally coloured specimens, from which he sent me a ♂ which is black on the upper side and shimmering bluish on the pronotum, and 2 ♀. Their legs are pitch-black, darkly rufous in the joints, or plain black. It is possible that this variety will also be found in Germany.

### ﻿*Plateumarisplanicollis* (Kunze, 1818)

Described as *Donaciaplanicollis* on page 34. Synonym of *P.rustica*.

**Table:** 

**Latin**	**English**
***Donaciaplanicollis***	***Donaciaplanicollis***
D.planicollis: thorace elongato, subquadrato, planiusculo, leviter impresso et punctato, postice angustato, margine nonnihil producto, lateribus subintegris, linea postica; elytris convexis, rugulosis, distincte punctato-striatis, apice rotundato, pedibus rufescentibus.	D.planicollis: the pronotum elongated, nearly quadrangular, rather flat, slightly impressed and punctured, narrowed posteriorly, the margin slightly convex, the sides reasonably complete, with a posterior line; the elytra arched, wrinkled, with distinct dotted stripes, rounded at the apex, the legs reddish.

### ﻿*Plateumarisroscida* Weise, 1912

Described on page 77.

**Table:** 

**Latin**	**English**
***Plateumarisroscida***	***Plateumarisroscida***
Elongata, subdepressa, supra obscuro-aenea, leviter aurichalceo-cupreo induta, subopaca, sericeo-micans, subtus argenteo-sericea, antennis pedibusque testaceo-variegatis;	Elongate, slightly flattened, upper side dark bronze-coloured, with thin brass-cupreous hairs, slightly dull, silky-lustrous, underside silvery-silky, antennae and legs patterned testaceous;
prothorace subquadrato, basin versus angustato, subtiliter ruguloso-punctato, elytris apice rotundato-truncatis, punctato-striatis, interstitiis dense subtilissime rugulosis, femoribus posticis dente valido armatis.	the prothorax almost square, narrowed towards the base, punctured finely and wrinkly, elytra at the apex roundly truncated, with dotted stripes, intervals densely and very finely wrinkled, hind femora armoured with a stout tooth.
Long. 7,5 mm.	Leng[th] 7.5 mm.
Transbaikalien: Dschitah (Ertl).	Transbaikalia: Chita ([donated by] Ertl).
**German**	**English**
Einer *Don.thalassina* Germ. ähnlich, aber neben *Plat.discolor* Panz. gehörig, von dieser und *sericea* L. durch gestreckten, viel flacheren Körper und die ziemlich matte Oberseite sofort zu unterscheiden.	Similar to *Don*[*acia*] *thalassina* Germ., but next to *Plat*[*eumaris*] *discolor* Panz., to be immediately distinguished from this one and *sericea* L. by the elongated, much flatter body and the rather dull upper side.
Dunkel metallisch braun, mit gelblichem Kupferschimmer, matt seidenartig glänzend, unterseits äußerst fein und dicht weißlich behaart,	Dark metallic brown, with a yellowish cupreous shimmer, matt silky shine, ventrally with extremely fine and dense whitish hairs,
Fühler und Beine dunkel rötlich gelbbraun, das erste Fühlerglied und die Spitze der folgenden Glieder mehr oder weniger weit schwärzlich, die obere Hälfte der Schenkel metallisch grünlich schwarz, die Spitze der Schienen und die Tarsen angedunkelt. Fühler schlank, Glied 3 länger als 2, 4 länger als 3.	antennae and legs dark reddish yellow-brown, the first antennal segment and the tip of the following segments more or less widely blackish, the upper half of the femora metallic greenish black, the apex of the tibiae and of the tarsi darkened. Antennae slender, segment 3 longer than 2, 4 longer than 3.
Thorax länger als breit, hinter dem heraustretenden vorderen Borstenkegel durch einen schwachen, schlecht begrenzten Seitenhöcker etwas erweitert, sodann eine Spur eingeschnürt, endlich bis zur Basis schwach verengt, auf der Scheibe sehr fein und dicht runzelig punktiert, mit einer feinen, verloschenen Mittelrinne. Diese erweitert und vertieft sich hinten und geht hier in einen Quereindruck über.	Thorax longer than wide, slightly widened behind the protruding anterior bristle cone by a weak, poorly defined lateral tubercle, then feebly constricted, finally weakly narrowed till to the base, very finely and densely wrinkled and punctate on the disc, with a fine, obliterated median groove, which widens and deepens towards the base and turns into a transverse impression there.
Flügeldecken äußerst dicht und fein querrunzelig, regelmäßig in Reihen punktiert, mit zwei verloschenen Eindrücken jederseits an der Naht.	Elytra wrinkled extremely densely and finely transversely, regularly punctured in rows, with two obliterated impressions on each side of the suture.

### ﻿*Plateumarisrustica* (Kunze, 1818)

Described as *Donaciarustica* on page 31.

**Table:** 

**Latin**	**English**
***Donaciarustica***	***Donaciarustica***
*D.rustica*: thorace subquadrato, depresso, subimpresso, vage et subtiliter punctato, angulis anticis rotundatis, lateribus integro, linea postica; elytris depressiusculis, subtiliter et obsolete punctato-striatis, interstitiis rugosis, coleopterorum apice rotundato; pedibus rufescentibus. a) Femoribus apice obscurioribus. mas. *D.rustica* Schüppel in litt. Lepturadiscolor Marsh. Ent. brit. I. 346; 14. fem. - fusca Marsh. l. c. 349. 20. mas. (secundum specimina a cel. Leachio missa.)	*D.rustica*: pronotum approximately square, flattened, slightly depressed, punctured sparsely and finely, front corners rounded, sides entire, with a posterior line; the elytra slightly flattened, finely and faintly streaked with dots, the interstices wrinkled, the apex of the elytra rounded; legs reddish. a) The femora darker at the posterior end. Male. *D.rustica* Schüppel in litt. Lepturadiscolor Marsh[am] Ent. brit. I. 346; 14. fem[ale] - fusca Marsh[m] l[oco] c[itato] 349. 20. Male. (according to the specimens sent by the known Leach.) [William Elford Leach (1790–1836), a then famous zoologist.]

### ﻿*Plateumarissachalinensis* Medvedev, 1973

Described on page 876. Synonym of *P.weisei*.

**Table:** 

**Russian**	**English**
***Plateumarissachalinensis***	***Plateumarissachalinensis***
Бронзово-зеленый или медный, переднеспинка красновто-медая, основания всех члеников усиков, а также основания бедер и голеней рыжпе, задние края стернитов брюшка с желто-рыжей окантовкой.	Bronze-green or cupreous, pronotum medium-reddish, bases of all antennal segments, as well as bases of femora and tibiae rufous, posterior margins of abdominal sternites with a yellow-rufous fringe.
Верх слабо блестящий, лобные бугры оченъ слабые, усики тонкие, длинные, как у *P.weisei* Duv., задние бедра с оченъ слабым, едва выступающим зубцом. Вершина пигидия и последний стернит брюшка притупленно округленные. Длина тела 6.6–7.1 mm. Эдеагус с зкозаостренной вершиной, снизу блестящий, в сглаженной продолъной морщинистости, парамеры значителъно не достигают вершины эдеагуса, с перетяжкой у основания.	Upper side slightly shining, frontal tubercles very weak, antennae thin, long, like in *P.weisei* Duv., hind femora with a very weak, barely protruding tooth. The apex of the pygidium and the last sternite of the abdomen are obtusely rounded. Body length 6.6–7.1 mm. Aedeagus with sharply pointed apex, shining ventrally, with smoothed longitudinal rugosity, parameres do not significantly reach the apex of the aedeagus and are constricted at the base.
Описываемый вид относится к номинативному подроду и наиболее близок к *P.obsoleta* Jacobs., от которого отличается двупветнй окраской ног. От *P.weisei* Duv. и *P.amurensis* Wse. отличается окраской ног и слабым зубцом на задних бедрах. Очевидно, вид занимает промжуточное положение между группой *P.sericea* и группой *P.weisei*. Надо скзатъ, что сравнение нового вида с *P.obsoleta* Jacobs., известного по единственной самке, затруднено тем, что в нашем распоряжении имелисъ толъко самцы.	The described species belongs to the nominative subgenus [*Plateumaris* was regarded as subgenus of *Donacia* by some authors] and is closest to *P.obsoleta* Jacobs. From *P.weisei* Duv. and *P.amurensis* Wse. it differs in the coloration of the legs and a weak tooth on the hind femora. Obviously, the species occupies an intermediate position between the *P.sericea* group and the *P.weisei* group. It must be said that the comparison of the new species with *P.obsoleta* Jacobs., known from a single female, is difficult, because we had only males at our disposal.
Сахалин: Южно-Сахалинск, 12 VII 1955 (голотип) и 10 VII 1955 (2 паратипа); сбор H.H. Филиппова.	Sakhalin: Yuzhno-Sakhalinsk, 12 VII 1955 (holotype) = and 10 VII 1955 (2 paratypes); collection H.H. Filippova.

### ﻿*Plateumarissericea* (Linnaeus, 1758)

Described as *Lepturasericea* on page 397. First description in Linnaeus (1758); description of additional characters in Linnaeus (1760) below.

**Table:** 

**Latin**	**English**
***Lepturasericea***	***Plateumarissericea***
INSECTACOLEOPTERA. Leptura.	INSECTACOLEOPTERA. Leptura.
180. LEPTURA. *Antennæ* setaceæ. *Elytra* apicem versus attenuata. *Thorax* teretiusculus.	180. LEPTURA. Antennae setaceous. Elytra narrowed toward the apex. Thorax terete [elongated and rounded].
*Thorace ovato s. antrorsum oblongiusculo angustiore. Elytris apice truncatis*.	Thorax egg-shaped or toward anterior part elongated-narrowed. Apex of the elytra truncated.
sericea. 5. L. viridi-cærulea, elytris subfastigiatis.	sericea. 5. green-blue L[eptura], with rather bevelled elytra.
*Habitat in* Europa.	Lives in Europe.

Description of additional characters on page 196.

**Table:** 

683. LEPTURA *sericea* Habitat apud nos rarius.	683. LEPTURA *sericea* [starts with the same text as in Linnaeus (1758) and additional:] Lives with us rarer. [with us = in Sweden]
*DESCR. Corpus* magnitudine Lept. aquaticæ, sed cæruleum, nitens totum. *Antennæ* nigræ vix corporis longitudine. *Thorax* quasi medius inter Lepturas & Cincindelas. *Elytra* punctato-striata.	*DESCR*[*IPTION*] Same size of the *body* as Lept[ura] aquatica, but blue, totally shiny. The black *antennae* scarcely as long as the body. The *thorax* more or less median between Leptura and Cicindela [species]. *Elytra* with dotted stripes.

### ﻿*Plateumarissibirica* (Solsky, 1871)

Remark: “Solsky, 1872” is wrong. For details see Geiser (2023).

Described as *Donaciasibirica* on page 245. Synonym of *P.sericea*.

**Table:** 

**Latin**	**English**
***Donaciasibirica***	***Donaciasibirica***
Oblongo-ovata, supra varicolor, viridi vel cupreo-metallica, subtus aureo-holosericea; prothorace elongato, angulis anterioribus acute extrorsum prominulis, utrinque antice tuberculato, supra planiusculo, basi leviter impresso, subtiliter coriaceo, obsolete canaliculato; elytris convexis, apice conjunctim rotundatis, vix impressis, punctato-striatis, interstitiis transversim rugosis; femoribus posterioribus subtus dente valido armatis. Long. 7–8 mlm.	Elongated-ovate, with varying colours above, green- or copper-metallic, golden-silky below; Prothorax elongated, the front angles pointed outwards, bulging on both sides ahead, almost flat at the top, slightly impressed at the base, finely leathery, with a faint groove; elytra convex, rounded together at apex, scarcely impressed, with dotted stripes, the interstices wrinkled across; hind femora reinforced with a strong tooth below. L[ength] 7–8 mm.
**French**	**English**
Voisine de la *D.sericea* L. à côté de laquelle elle doit aussi prendre place; elle lui ressemble par son habitus général, la forme et la sculpture des élytres, mais elle est plus petite, moins obese, à antennes plus grèles et plus allongées; les mandibules, sauf la base et l’extrémité sont ainsi que le palpes, moins l’extrémité des articles, d’un rouge ‘ferrugineux. La tête, l’écusson et tout le dessous sont couverts d’une fine pubescence soyeuse dorée, le corselet et les élytres en dessus tantôt cuivreux tantôt d’un vert bronzé.	Closely related to *D.sericea* L., next to which it must also take place; it resembles it by its general habitus, the shape and the sculpture of the elytra, but it is smaller, less obese, with more green and more elongated antennae; the mandibles except the base and the apex, as well as the palps except the apex of the joints, are ferruginous red. The head, the scutellum and all the underside are covered with fine golden silky hairs, the pronotum and the elytra above sometimes coppery, sometimes brown-green.
La tête plus finement chagrinée que chez *D.sericea* avec sillon frontal plus faible; les yeux plus globuleux et plus saillants, l’étranglement postoculaire plus fort. Les articles des antennes allongés, 3 plus long que 2, mais plus court que les suivants.	The head more finely shagreened than in *D.sericea* with a weaker frontal groove; eyes more protruding and more prominent, with a stronger postocular constriction. The antennomeres are elongated, the 3^rd^ one longer than the 2^nd^ one, but shorter than the following ones.
Le corselet comme chez *D.sericea* L., mais avec les angles antérieurs très pointus et saillants en dehors; sa surface est plus densement et plus finement rugueuse, subopaque, avec un très faible sillon au milieu et faiblement impressionnée près de la base. Les points des tries des élytres sont plus serrés, le stries elles mêmes plus profondes, les intervalles plus convexes et beaucoup plus fortement transversalement rides.	The pronotum as in *D.sericea* L., but with the very pointed anterior angles protruding outside; its surface is more densely and more finely rough, subopaque, with a very weak groove in the middle and weakly impressed near the base. The punctures of the rows on the elytra are more densely packed, the stripes themselves deeper, the interstices more convex and much more strongly wrinkled transversely.
Irktsk, VII.	Irkutsk, VII. [July? The meaning of “VII.” is unclear; no date or even year of collection given in the first description.]

### ﻿*Plateumarissulcifrons* Weise, 1900

Described on page 267. Synonym of *P.rustica*.

**Table:** 

**Latin**	**English**
***Plateumarissulcifrons*** ♀:	***Plateumarissulcifrons*** ♀:
Oblonga, convexiuscula, supra aenea, subtus piceo-nigra, griseo-sericea, antennis, tibiis tarsisque obscure rufescentibus, fronte late et profunde sulcata, prothorace quadrato, subtilissime pubescente, ante basin obsolete constricto, disco planiusculo, nitido, sat crebre punctato, canalicula media antice posticeque profundiore impresso, tuberculo obsoleto utrinque, subpolito, elytris punctato-striatis, interstitiis transversim strigosis, femoribus inermibus.	Long, slightly convex, bronze coloured above, below jet-black, silky-grey, the antennae, tibiae and tarsi dark reddish, the frons with a wide and deep groove, the prothorax square, very finely pubescent, very slightly constricted before the base, disc almost flat, shiny, rather densely punctured, the impressed middle groove deepened ahead and behind, the tubercles on both sides hardly perceptible, slightly smoothed, the elytra with dotted stripes, intervals narrow in width, the femora unarmed.
Long. 8–9 mm.	Leng[th] 8–9 mm
Zeitun (Staudinger).	Zeitun ([leg.] Staudinger).
Var. a. Antennis pedibusque testaceis.	Var[iation] a. Antennae and legs testaceous.
**German**	**English**
Der *Plat.rustica* und *affinis* ähnlich, neben letztere zu stellen, gestreckter als beide, durch die tiefe und breite Stirnfurche, deren Seiten hohe Längswülste bilden, das glänzende Halsschild, dessen beiderseits abgekürzte Mittelrinne am Anfange und Ende tief und scharf, in der Mitte flach ist und neben der sich jederseits ein sehr flacher, spiegelglatter Höcker befindet, sowie die völlig ungezähnten Hinterschenkel sicher verschieden.	Similar to *Plat.rustica* and *affinis*, to be placed next to the latter, more elongated than both, certainly different due to the deep and broad frontal furrow, whose sides form high longitudinal ridges, the shiny pronotum, whose median groove, which is shortened on both sides, is deep and sharp at the beginning and the end, flat in the middle, and next to which there is a very flat, mirror-smooth tubercle on each side, as well as the completely unarmed hind femora.

### ﻿*Plateumaristenuicornis* Balthasar, 1934

Described as Plateumaris (Juliusina) tenuicornis on page 128. Synonym of *P.consimilis*.

**Table:** 

**German**	**English**
***Plateumaristenuicornis***	***Plateumaristenuicornis***
Der *Pl.consimilis* Schrank äußerst nahestehende Art, mit ihr auch wahrscheinlich bisher vermengt.	A species very closely related to *Pl.consimilis* Schrank, with which it has also probably been confused till now.
Kopf sehr dicht und fein punktiert, mit einer scharfen, länglichen Rinne, die Erhabenheiten an der Wurzel der Fühler nur mäßig, die Augen sehr stark vorgequollen, die Schläfen stark entwickelt, mächtig hervorragend, nach hinten deutlich konvergierend. Die Halspartie stark eingeschnürt, daher auffällig akzentiert. Fühler sehr schlank, die einzelnen Glieder zur Spitze nur mäßig verstärkt.	Head punctured very densely and finely, with a sharp, elongated groove, the elevations at the base of the antennae are only moderate, the eyes are very protruding, the temples are well developed, powerfully prominent, clearly converging towards the base. The neck area is severely constricted, therefore conspicuously accentuated. Antennae very slender, the individual segments only moderately widened towards the tip.
Halsschild mit einer ziemlich deutlich angedeuteten Mittelfurche, auf der Scheibe nicht besonders dicht, aber ziemlich fein punktiert, dazwischen äußerst fein chagriniert, nur an den Seiten mit kaum wahrnehmbaren anliegenden Härchen (erst bei der Vergrößerung, 40×, Zeiß, binokulares Mikroskop) besetzt. Die Seitenbeulen ziemlich stark entwickelt, die Seiten nach hinten stärker zusammenlaufend, die Vorderwinkel spitzig vorragend.	Pronotum with a fairly clearly indicated central furrow, punctured on the disc not particularly densely, but quite finely, extremely finely shagreened in between, only on the sides overgrown with barely perceptible flat small hairs (only by magnification, 40×, Zeiss, binocular microscope). The side tubercles fairly well developed, the sides tapering more towards the end, the front angles pointedly prominent.
Flügeldecken ziemlich fein in Längsreihen punktiert, die Zwischen- räume sehr fein und sehr dicht quergerunzelt, zur Basis vollkommen flach, gegen die Spitze mäßig gewölbt. Unterseite seidenglänzend, dicht, kurz, anliegend behaart.	Elytra rather finely punctured in longitudinal rows, the intervals very finely and very densely wrinkled transversely, completely flat at the base, moderately convex towards the apex. Underside silky shiny, dense, short, hairy.
Hinterschenkel nur mit kleinem Zähnchen (beim ♀), die Schienen verhältnismäßig schlank.	Hind femora only with small teeth (♀), the tibiae relatively slender.
L. 6.5 mm	L[ength] 6.5 mm
Oberseite grün, ziemlich matt erscheinend, Beine und Fühler hell gelbrot.	Upper surface green, appearing rather dull, legs and antennae pale yellow-red.
Bosnien, Dol. Tuzla, Em. Fritsch leg.	Bosnien, Dol[na] Tuzla, Em[merich] Fritsch leg.
Von der sehr verwandten Art *Pl.consimilis* Schrank durch folgende Merkmale ziemlich schwer, aber sicher zu unterscheiden: Fühler auffallend schlanker, Augen sehr stark vorgequollen, die Schläfen nach hinten konvergierend, nicht parallel und viel mehr akzentiert, Hals sehr deutlich stärker eingeschnürt, schmäler, die Lateralbeulen des Halsschildes deutlicher, oben tiefer abgegrenzt, der Halsschild schmäler, nach hinten stärker zusammenlaufend. Außerdem sind die Vorderwinkel mehr seitlich gerichtet und spitziger. Die Flügeldecken bei der neuen Art (im Falle, daß die Skulptur vollkommen konstant ist) scheinen viel feiner und dichter skulptiert zu sein. Im ganzen subtiler gebaut und kleiner.	Rather difficult to distinguish but surely from the very related species *Pl.consimilis* Schrank by the following characters: antennae noticeably slimmer, eyes very much bulging, temples converging backwards, not parallel and much more accentuated, neck much more constricted, narrower, the lateral bulges of the pronotum more distinct, more deeply demarcated above, the pronotum narrower, converging more towards the end. In addition, the front angles are more laterally directed and more pointed. The elytra in the new species (in case the sculpture is perfectly constant) appear to be much finer and more densely sculptured. On the whole built more subtly and smaller.

Bechyné (1942) wrote in a paragraph that *Plateumaristenuicornis* Balthasar looks the same as *P.consimilis*. This article was ignored for decades, perhaps because it was printed in Czech and Latin. Here the part of that article that deals with *P.tenuicornis* in Latin is copied and translated into English.

**Table:** 

**Latin**	**English**
**De variatione Plateumarisconsimilis Schrank. (Col. Donaciidae.)**	**About the variability of Plateumarisconsimilis Schrank. (Col. Donaciidae.)**
In opusculo hoc recensionem *Plateumaristenuicornis* Balthasar, Entom. Nachrichtenbl. VIII, 1934, pp. 128–129 affero. Secundum exempl. quae ante oculos habeo, *Pl.consimilis* et *Pl.tenuicornis* haud specifice differunt. Variat enim *Pl.consimilis* in characteribus sequentibus: in longitudine corporis, in convexione oculorum, in forma temporum, in forma singulorum articulorum antennarum, in punctatione capitis, prothoracis elytrorumque nec non in forma angulorum anticorum pronoti, in dentibus femorum posteriorum etiamque in colore species haec maximam variationem demonstrat.	In this work I provide a review of *Plateumaristenuicornis* Balthasar, Entom. Newsletter VIII, 1934, pp. 128–129. According to the specimens on hand, there are no typal differences between *Pl.consimilis* and *Pl.tenuicornis*. That is to say *Pl.consimilis* varies in the following characters: In the length of the body, in the curvature of the eyes, in the shape of the temples, in the shape of the individual antennal segments, in the dotting of the head, the prothorax and the elytra and also in the shape the front angles of the pronotum, in the teeth of the hind femora and also in the colouring this species shows a very large variability.

### ﻿*Plateumarisweisei* (Duvivier, 1885)

Described as Donacia (Plateumaris) weisei on page cxvi [116].

**Table:** 

**French**	**English**
***Donaciaweisei***	***Donaciaweisei***
Corps oblong, rétréci en arrière, d’un bronzé verdâtre ou d’un violet foncé, couvert en dessous d’une pubescence d’un gris argenté assez dense, avec les pattes, les antennes et la bouche ferrugineuses; corselet allongé, presque plan en dessus, très-rétréci en arrière, sillonné à la base, densément ponctué; élytres allongées, ponctuées-striées, à intervalles ridés transversalement, peu convexes, rétrécies en arrière.	Body elongated, back narrowed, greenish-brown or dark purple, ventrally covered with a fairly dense silvery-grey pubescence, legs, antennae and mouth rufous; pronotum elongated, almost flat above, very narrow behind, furrowed at the base, densely punctured; elytra elongated, with punctured stripes, at the intervals transversely wrinkled, not very convex, narrowed behind.
♂ Plus petit, étroit, allongé; antennes des ^2^/_3_ de la longueur du corps. ♀ Plus grande, large, robuste; antennes de la ½ de la longueur du corps. Long. 7 to 7 ¾ mill. – Sibérie (H. Deyrolle).	♂ Smaller, narrow, elongated; antennae ^2^/_3_ the length of the body. ♀ larger, broad, sturdy; antennae ½ the length of the body. L[ength] 7 to 7 ¾ mm – Siberia (H. Deyrolle).
♂ Corps allongé, étroit, rétréci en arrière, d’un beau vert bronzé ou d’un violet foncé en dessus. Bouche ferrugineuse. Tête marquée d’un profond sillon longitudinal plus ou moins prolongé en arrière, finement rugueuse, profondément ponctuée. Yeux noirs, assez saillants.	♂ Body elongated, narrow, back narrowed, of a beautiful bronze-green or dark purple above. Mouth rufous. Head marked with a deep longitudinal groove more or less long, finely rugose, deeply punctured. Black eyes, rather prominent.
Antennes des ^2^/_5_ de la longueur du corps, ferrugineuses, à art. 3–4 subégaux, le 3^e^ (= third) cependant un peu plus court et plus robuste que le suivant. Prothorax un peu plus long que son plus grand diamètre transversal et assez fortement rétréci en arrière; coupé carrément en avant, légèrement arrondi et subsinué à la base, à angles peu distincts; angles antérieurs suivis en arrière d’un renflement arrondi, occupant les ^2^/_5_ des côtés, nettement limité en dessous par une dépression assez marquée provoquant en cet endroit un rétrécissement du corselet; disque presque plan, couvert de points, enfoncés, très-serrés et confluents, sans sillon dorsal, ayant, près de la base, un sillon transversal anguleux n’atteignant pas les côtés.	Antennae ^2^/_5_ of body length, rufous, with antennomeres 3–4 nearly equal, the 3^rd^ one however a little shorter and more robust than the next one. Prothorax slightly longer than its greatest transversal diameter and quite fiercely narrowed posteriorly; cut squarely in front, slightly rounded and emarginate at the base, with slightly distinct angles; anterior angles followed behind by a rounded swelling, occupying ^2^/_5_ of the sides, clearly limited below by a fairly marked depression causing in this spot a narrowing of the pronotum; disc almost flat, covered with dots, sunken, very close and confluent, without a dorsal groove, near the base with an angular transverse groove not reaching the sides.
Ecusson en triangle curviligne, ponctué. Elytres oblongues, très-rétrécies en arrière, finement ponctuées-striées, à points très rapprochés; intervalles entre les stries à peine relevés, couverts de fines rugosités transversales. Dessous coloré comme le dessus, avec l’extrémité abdominale ferrugineuse, couvert d’une pubescence d’un gris argenté assez dense. Pattes ferrugineuses, cuisses postérieures légèrement renflées, munies près de leur extrémité d’une dent triangulaire de dimension variable.	Scutellum punctured. Elytra elongated, distinctly narrowed behind, finely punctured-striated, with very close dots; intervals between the stripes barely raised, covered with fine transverse wrinkles. Underside coloured like above, with a rufous abdominal apex, covered with a fairly dense silvery grey pubescence. Legs rufous, hind femora slightly bulging, provided near to their end with a triangular tooth of variable size.
♀ Forme plus robuste, corselet relativement plus large, antennes n’atteignant que la moitié de la longueur du corps.	♀ Shape more robust, pronotum relatively wider, antennae reaching only half the length of the body.
Cette espèce est très-voisine de *D.discolor* Hoppe; elle en diffère par sa forme plus svelte, plus étroite, sa taille plus petite, son corselet plus allongé, plus rétréci en arrière, avec ses côtés non régulièrement arrondis mais renflés légèrement sur le premier tiers, par l’absence de sillon longitudinal sur le prothorax, par ses élytres plus étroites et la dent triangulaire des fémurs postérieurs moins saillante.	This species is closely related to *D.discolor* Hoppe [= synonymous with *Plateumarisconsimilis* Schrank]; it differs from it by its more slender, narrower shape, its smaller size, its more elongated pronotum which is more narrowed behind, with its sides not regularly rounded but slightly swollen on the first third, by the absence of a longitudinal groove on the prothorax, by its narrower elytra and the less prominent triangular tooth of the hind femora.
Je dédie cette espèce à notre collègue, M. Julius Weise de Berlin, à qui la science entomologique est redevable de sérieux travaux sur les Phytophages.	I dedicate this species to our colleague, Mr. Julius Weise from Berlin, to whom entomological science is indebted for serious work on Phytophages.

## ﻿Discussion

There are ten Palaearctic *Plateumaris* species regarded as valid: three of them were described in English (*P.akiensis*, *P.constricticollis*, *P.shirahatai*), six in Latin (*P.amurensis*, *P.bracata*, *P.consimilis*, *P.roscida*, *P.rustica*, *P.sericea*), and one (*P.weisei*) was originally described in French. In general, most of the original descriptions of *Plateumaris* taxa are in Latin or began with a Latin diagnosis at least, and further explanations were then added in German, Russian, or French in most cases.

In addition to the first descriptions of the valid species in the Palaearctic, another 19 original descriptions are presented here with their translations and the species names published therein are now regarded as synonyms. Mostly, these are names which were synonymized or their synonymisation was confirmed in Geiser (2023). This list is not complete, because more than 70 names are now known to be allocated to one of the ten valid *Plateumaris* species. We intend to continue publishing translations of original descriptions of Donaciinae taxa. Furthermore, we encourage other colleagues to do the same in their areas of expertise.

The Latin first description of the genus *Plateumaris* established by Thomson (1859) and its translation are also given here. At first, Donaciinae species were assigned to the genus *Leptura* by Linnaeus (1758). Later, Fabricius (1775) established the genus *Donacia*, but did not change the genus name of *Lepturasericea*. Later, some authors described *Plateumaris* species with *Donacia* as the genus name, even after Thomson had established the name *Plateumaris*. Some authors regarded Plateumaris only as a subgenus of Donacia, and these opinions are reflected in the first descriptions. For more details see Geiser (2023).

## ﻿Additional information

### Author ORCIDs

Elisabeth Geiser https://orcid.org/0009-0005-4536-8938

### Data availability

All of the data that support the findings of this study are available in the main text or Supplementary Information.

## References

[B1] Apfelbeck V (1912) Komponente balkanske faune iz roda Chrysomelidae. Zoogeografski prinos fauni balkanskog Poluostrova. Glasnik Zemaljskog Muzeja u Bosni i Hercegovini 24: 235–263.

[B2] Balthasar V (1934) Ein Beitrag zur Kenntnis der palaearktischen Donaciini. Entomologisches Nachrichtenblatt (Troppau) 8(4): 128–130. https://www.zobodat.at/publikation_volumes.php?id=62568

[B3] Bechyné J (1942) O variabilitě *Plateumarisconsimilis* Schrank. (Col. Donaciidae). De variatione *Plateumarisconsimilis* Schrank. (Col. Donaciidae). Sborník entomologickèho odděleni zemědělské Musea v Praze (= Acta Entomologica Musei Nationalis Pragae) 20: 232–237. [in Czech and Latin] https://www.aemnp.eu/acta-entomologica/volume-20/482/o-variabilite-plateumaris-consimilis-schrank-col-donaciidae-de-variatione-plateumaris-consimilis-schrank-col-donaciidae.html

[B4] Bousquet Y (2016) Litteratura Coleopterologica (1758–1900): A guide to selected books related to the taxonomy of Coleoptera with publication dates and notes. ZooKeys 583: 1–776. 10.3897/zookeys.583.7084

[B5] Clavareau H (1913) Chrysomelidae: l. Sagrinae, 2. Donaciinae, 3. Orsodacninae, 4. Criocerinae. In: Junk W Schenkling S (Eds) Coleopterorum Catalogus auspiciis et auxilio.Junk W, Berlin Pars 51: 3–115.

[B6] Duvivier A (1885) Quatre Phytophages nouveaux. Bulletin ou Comptes-Rendus des Scéances de la Société Entomologique de Belgique, 1885: cxvi–cxix. https://www.biodiversitylibrary.org/item/303035#page/120/mode/1up

[B7] Fabricius JC (1775) Systema entomologiae sistens insectorum classes, ordines, genera, species, adiectis synonymis, locis, descriptionibus, observationibus. Flensburgi et Lipsiae, Libraria Kortii, 832 pp. 10.5962/bhl.title.36510

[B8] Fabricius JC (1792) Entomologia systematica emendata et aucta, secundum classes, ordines, genera, species, adjectis, synonimis, locis, observationibus, descriptionibus. Tomus I. Pars II. Hafniae: C. G. Proft, 538 pp. 10.5962/bhl.title.125869

[B9] Geiser E (2019) To be or not to be a synonym – revision of the *Donaciaclavareaui-fukiensis* complex (Coleoptera, Chrysomelidae, Donaciinae). In: Schmitt M Chaboo CS Biondi M (Eds) Research on Chrysomelidae 8.ZooKeys 856: 27–50. 10.3897/zookeys.856.32388 31258367 PMC6591214

[B10] Geiser E (2023) Revision of the Palaearctic species of the genus *Plateumaris* C. G. Thomson, 1859 (Coleoptera: Chrysomelidae: Donaciinae). In: Schmitt M Chaboo CS (Eds) Research on Chrysomelidae 9.ARPHA Preprints, 1–36. 10.3897/arphapreprints.e103443 PMC10483391 37692324

[B11] Geiser E (in press) Donaciinae. In: Bezděk J, Sekerka L (Eds) Catalogue of Palaearctic Coleoptera (Vol. 6/2(. Revised and updated Second Edition. Chrysomeloidea II (Megalopodidae, Orsodacnidae, Chrysomelidae). Brill, Leiden.

[B12] Geiser E Bezděk J (in press) New Nomenclatural and Taxonomic Acts, Comments and Distribution: Donaciinae. In: Bezděk J, Sekerka L (Eds) Catalogue of Palaearctic Coleoptera (Vol. 6/2). Revised and updated Second Edition. Chrysomeloidea II (Megalopodidae, Orsodacnidae, Chrysomelidae). Brill, Leiden.

[B13] Geiser E Jäch MA (2021) Explanatory notes on the updates concerning the genus *Donacia* Fabricius, 1775 in the second edition of the Catalogue of Palaearctic Coleoptera (Vol. 6/2) (Coleoptera: Chrysomelidae). Koleopterologische Rundschau 91: 155–178. https://www.zobodat.at/publikation_series.php

[B14] Jacobsohn G (1892) Analytische Übersicht der bekannten *Donacia*- und *Plateumaris*-Arten der Alten Welt. Horae Societatis Entomologicae Rossicae 26: 412–437.

[B15] Jacobsohn [= Jacobson] GG (1894) Adnotationes de Chrysomelidis nonnullis novis vel parum cognitis. Horae Societatis Entomologicae Rossicae 28: 242–246.

[B16] Jacoby M (1885) Descriptions of the phytophagous Coleoptera of Japan, obtained by Mr. George Lewis during his second journey, from February 1880 – September 1881. Part I. Proceedings of the scientific meetings of the Zoological society of London 1885: 190–211. [pl. XI] 10.1111/j.1096-3642.1885.tb02894.x

[B17] Kimoto S (1971) [new taxa]. In: Kimoto S Hiura I (Eds) List of the Chrysomelidae specimen preserved in the Osaka Museum of Natural History, III. (Insecta: Coleoptera).Bulletin of the Osaka Museum of Natural History 25: 1–26. 10.5962/bhl.part.19678

[B18] Kolossow J (1930) Annotationes de quibusdam Donaciini. Coleopterologisches Zentralblatt 5: 28–29.

[B19] Kunze G (1818) Beiträge zur Monographie der Rohrkäfer. Neue Schriften der Naturforschenden Gesellschaft zu Halle 2(4): 1–56.

[B20] LeGrand G (1861a) Liste des coléoptères du département de l’Aube. Mémoires de la Société Académique d’Agriculture, des Sciences, Arts et Belles-Lettres du Département de l’Aube, Deuxième Série 12: 181–274. [DP: 13 July 1861] [Bibl. France 1861, 50 (28): 338]

[B21] Linnaeus C (1758) Systema Naturae per Regna Tria Naturae, Secundum Classes, Ordines, Genera, Species, cum Characteribus, Differentiis, Synonymies, Locis. Tomus I. Editio decima, reformata. Holmiae: Impensis Direct. Laurentii Salvii, [iv +] 824 [+ 1] pp. 10.5962/bhl.title.542

[B22] Linnaeus C (1760) Fauna Suecica Sistens Animalia Sueciae Regni: Mammalia, Aves, Amphibia, Pisces, Insecta, Vermes. Distributa per Classes et Ordines, Genera et Species (2^nd^ edn.). Laur, Salvii, Stockholmiae, 578 pp. https://www.biodiversitylibrary.org/item/100333#page/256/mode/1up

[B23] Medvedev LN (1973) Novye zhuki-listoedy (Coleoptera, Chrysomelidae) palearktiki [New leaf-beetles (Coleoptera, Chrysomelidae) from Palaearctic]. Entomologicheskoe Obozrenie 52: 876–885.

[B24] Panzer GWF (1795) Entomologia Germanica exhibens insecta per Germaniam indigena secundum classes, ordines, genera, species adiectis synonymis, locis, observationibus. I. Eleuterata. Norimbergae: Felssecker, [8] + 12 + 370 + 2 pp. [12 pls.] [DP: 14 February 1795] [Bousquet 2016] https://www.biodiversitylibrary.org/item/44240#page/282/mode/1up

[B25] Perris E (1864) Description de quelques espèces nouvelles de coléoptères et notes diverses. Annales de la Société Entomologique de France 4(4): 275–310.

[B26] Redikortsev VV (1908) Materials to the entomofauna of the Urals. Notes of Ural society of lovers of natural history 27: 95–122. [in Russian]

[B27] Reitter E (1920) Bestimmungstabellen der europäischen Coleopteren. Heft 88: Chrysomelidae, 1.Teil: Tribus Donaciinae. Wiener Entomologische Zeitung 38(1–3): 21–43.

[B28] Schrank F de Paula (1781) Enumeratio insectorum Austriae indigenorum. Augustae Vindelicorum: E. Klett et Franck, [24 +] 548 [+ 4] pp. https://www.zobodat.at/publikation_volumes.php?id=34990

[B29] Scopoli JA (1772) Observationes Zoologicae. Annus V. Historico-Naturalis. Lipsiae: Christ. Gottlob Hilscheri, 70–128.

[B30] Semenov AP (1895) Coleoptera asiatica nova. V. Horae Societatis Entomologicae Rossicae 29[1894–1895]: 251–270. https://www.zobodat.at>pdf>WEZ_14_0276

[B31] Shavrov WB (1948) Novye formy i mestonakhozhdeniya donaciy (Coleoptera, Chrysomelidae, Subfam. Donaciinae) fauny SSSR. II. Byulleten’ Moskovskogo Obshchestva Ispytateley Prirody, Otdel biologicheskiy. (N. S.) 53(1): 49–52. [New forms and localities of Donacia (Coleoptera, Chrysomelidae, Subfam. Donaciinae) of the fauna of the USSR. II Bulletin of the Moscow Society of Natural Scientists, Biological Department. (N. S.) 53(1): 49–52.]18861020

[B32] Silfverberg H (2010) Donaciinae. In: Löbl I Smetana A (Eds) Catalogue of Palaearctic Coleoptera (Vol.6). Apollo Books, Stenstrup, 354–368.

[B33] Solsky SM (1871) Coleopteres de la Siberie orientale. Horae Societatis Entomologicae Rossiae 8[1871–1872]: 232–277.

[B34] Thomson CG (1859) Skandinaviens Coleoptera, synoptiskt bearbetade. Tom. I. Lundbergska Boktryckeriet, Lund, 290 pp. 10.5962/bhl.title.138677

[B35] Tominaga O Katsura K (1984) Studies of the Japanese Donaciinae (Coleoptera: Chrysomelidae). 2. Notes on geographical diversity of *Plateumarisconstricticollis*, with description of an allied new species. Bulletin of the Osaka Museum of Natural History 37: 25–40.

[B36] Weise J (1893) Chrysomelidae. Lieferung 6. Naturgeschichte der Insecten Deutschland. Erste Abtheilung Coleoptera. Sechster Band [1893]. Nicolaische Verlags-Buchhandlung R. Stricker, Berlin, 1161 pp. [1 pl.] https://www.biodiversitylibrary.org/item/105313#page/69/mode/1up

[B37] Weise J (1898) Ueber neue und bekannte Chrysomeliden. Archiv für Naturgeschichte 64(1): 177–224. https://www.zobodat.at/publikation_series.php

[B38] Weise J (1900) Beschreibungen von Chrysomeliden und synonymische Bemerkungen. Archiv für Naturgeschichte 66(1): 267–296. https://www.zobodat.at/publikation_series.php

[B39] Weise J (1912) Beitrag zur Kenntnis der Chrysomeliden. Archiv für Naturgeschichte 78(A2): 76–98. https://www.zobodat.at/publikation_series.php

[B40] Zaitzev FA (1930) K rasprostraneniyu na Kavkaze vidov triby Donaciini (Coleoptera, Chrysomelidae). Bulletin du Musée de GeoPedriliargie 5: 105–114.

